# The Performance Analysis of Distributed Brillouin Corrosion Sensors for Steel Reinforced Concrete Structures

**DOI:** 10.3390/s140100431

**Published:** 2013-12-27

**Authors:** Heming Wei, Xuefeng Zhao, Xianglong Kong, Pinglei Zhang, Yanjun Cui, Changsen Sun

**Affiliations:** 1 College of Physics and Optoelectronic Engineering, Dalian University of Technology, Dalian 116024, China; E-Mails: hmwei@mail.dlut.edu.cn (H.W.); zpl200421013@gmail.com (P.Z.); 2 Department of Civil Engineering, Dalian University of Technology, Dalian 116024, China; E-Mail: cuiyanjun863@163.com; 3 Power Plant Life Management Research Center, Suzhou Nuclear Power Research Institute, Suzhou 215004, China; E-Mail: kongxianglong@cgnpc.com.cn

**Keywords:** corrosion monitoring, reinforced concrete structures, low-coherent fiber-optic strain sensor, BOTDA

## Abstract

The Brillouin optical time-domain analysis (BOTDA)-based optical fiber method has been proposed to measure strain variations caused by corrosion expansion. Spatial resolutions of 1 m can be achieved with this kind of Brillouin sensor for detecting the distributed strain. However, when the sensing fiber is wound around the steel rebar in a number of circles in a range of several meters, this spatial resolution still has limitations for corrosion monitoring. Here, we employed a low-coherent fiber-optic strain sensor (LCFS) to survey the performance of Brillouin sensors based on the fact that the deformation measured by the LCFS equals the integral of the strains obtained from Brillouin sensors. An electrochemical accelerated corrosion experiment was carried out and the corrosion expansion was monitored by both BOTDA and the LCFS. Results demonstrated that the BOTDA can only measure the expansion strain of about 1,000 με, which was generated by the 18 mm steel rebar corrosion, but, the LCFS had high sensitivity from the beginning of corrosion to the destruction of the structure, and no obvious difference in expansion speed was observed during the acceleration stage of the corrosion developed in the reinforced concrete (RC) specimens. These results proved that the BOTDA method could only be employed to monitor the corrosion inside the structure in the early stage.

## 1. Introduction

Steel corrosion is one of the most important durability issues in reinforced concrete (RC) structures because chlorides permeate concrete and corrode steel rebar, consequently accelerating the destruction of a structure, especially in marine environments [1]. Corrosion deteriorates the strength of structures, and leads to the risk of collapse. Thus, monitoring the corrosion condition of steel in RC structures is necessary.

In principle, the corrosion of steel in concrete is a typical electrochemical process that occurs slowly in a natural environment. Thus, the mainly researched method for this phenomenon is the simulation of a real corrosion environment by an electrochemical accelerated corrosion experiment under laboratory conditions [2]. Many techniques have been proposed for detecting corrosion in concrete, such as electrochemical techniques, ultrasonic, and resistance methods. Duffó *et al.* [3] developed an embeddable sensor with different electrodes for monitoring corrosion processes in new and existing RC structures. Lu *et al.* [4] designed an electrical corrosion sensor with an embeddable reference electrode combined with a linear polarization resistance method and electrochemical impedance spectroscopy for monitoring heterogeneous corrosion. Dong *et al.* [5] proposed a multifunctional sensor for detecting the PH and Cl^−^ concentration in concrete *in situ*. The sensor can also be used for determining the corrosion behavior of reinforcing steel in concrete. The authors found that corrosion of reinforcing steel occurs after 124 days of exposure, corresponding to a distinct increase in Cl^−^ concentration and decrease in pH. However, several holes must be drilled in old concrete constructions when utilizing this sensor.

Although electrochemical sensors can provide more and earlier *in situ* information to reflect the corrosion process of concrete structures, the corrosion of the electrodes themselves limits the durability of the sensors, which is still a key problem for long time monitoring. Kawasaki *et al.* [6] utilized acoustic emission to identify the onset of corrosion in rebar and the nucleation of concrete cracks in a cyclic wet-dry test. However, the received acoustic emission signals were affected by the thickness of the outer layer and the environment, thus obtaining valid data for durable monitoring was difficult.

Recently, researchers have proposed a method based on optical time-domain reflectometry (OTDR) for monitoring the corrosion of steel rebar in concrete structures [7]. Each sensor head consists of an optical fiber having a cleaved end coated with an aluminum film, which thickness becomes thinner as the corrosion becomes more severe. Thus, the light leak outside of fiber core and the attenuation of the reflected signal is increased. This method can monitor several specimens simultaneously by relative changes of the reflected signal gain to reflect the progress of corrosion. However, the method is qualitative in monitoring the tendency of corrosion, and cannot evaluate the corrosion quantitatively. The OTDR feature is dependent on the intensity of the reflected signal, which limits the long term stability of the demodulation signal. In addition, the durability of the sensors needs to be solved. Among the recently relative methods based on optical fiber sensors, Brillouin optical time-domain analysis (BOTDA) distributed sensors are more feasible for realizing durability monitoring [8], in which a sensing fiber is wound around the steel rebar to be monitored and the expansion caused by corrosion is monitored to reflect the process. BOTDA can provide a local and distributed strain that can reflect the expansion induced by corrosion. However, the spatial resolution limits the precision of BOTDA, which is defined as the smallest segment capable of discerning the average strain or temperature along the optical fiber length. Spatial resolution is determined by the duration of the incident optical pulse, where a 10 ns width corresponds to 1 m spatial resolution along the fiber. The accuracy of the measurements depends on the length and the uniformity of the strain or temperature within this segment. Thus, it is necessary to develop a method to judge the performance of this kind of wound sensor.

The present study introduces a direct quantitative method for survey the performance of the BOTDA corrosion expansion sensors by LCFS, which is a long term gauge sensor that has the potential for long time monitoring the corrosion of steel in RC structures [9]. LCFS can directly obtain the total deformation, whereas BOTDA can obtain the distributed stain along the fiber, which can be integrated over the sensing fiber to estimate the deformation resolving capability of the sensor. The results demonstrated that the BOTDA sensor has an advantage in multipoint measurements, but a weaker signal is detected when more serious corrosion progressed. However, when the BOTDA sensor was out of use, the LCFS can still work. The fact that LCFS can find the initiation stage and the acceleration stage under the laboratory environment provides a potential method for studying the steel corrosion process and crack development in RC structures.

## 2. Theory Background

### 2.1. Principle of LCFS

**Figure 1.**
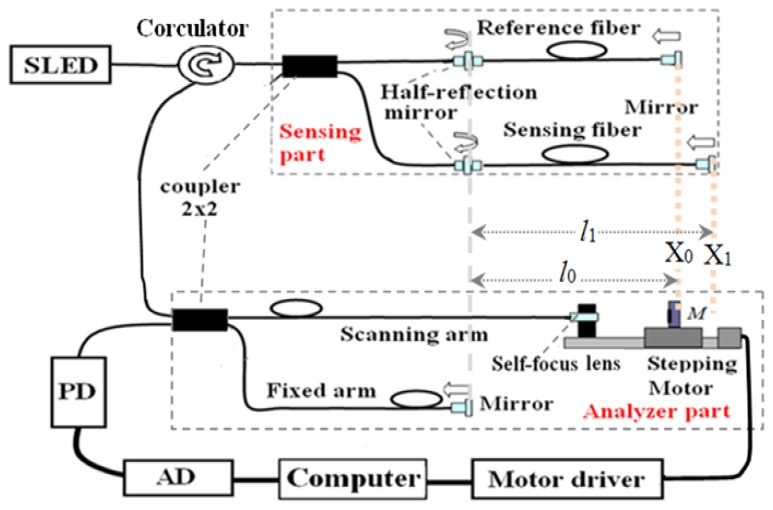
Configuration of the LCFS system.

The LCFS system is utilized for the measurement of steel corrosion expansion in concrete as shown in Figure 1. This principle, in order to get rid of the fluctuation of the introduction fiber by introducing an analyzer part, was firstly proposed in [10] and thenused in the practical tests [9,11]. Here we changed the coupler into a circulator and improved the system performance by using a reference fiber. The light comes from a super-luminescent emitting diode (SLED) source, with a center wavelength of 1,310 nm and a bandwidth of 45 nm, through a circulator coupled into two arms of a 3 dB coupler. The light of one arm is transmitted through a half-reflection mirror to a reference fiber and reflected by a mirror. The light of the other arm is also transmitted through a half-reflection mirror to the sensing fiber and reflected by a mirror. The light reflected by the half mirror and the mirror in both the reference and sensing fiber are coupled into the coupler again, and then through the circulator connected to a 3 dB 2 × 2 coupler. The light of one arm is transformed into a parallel light by a self-focusing lens, projected to a scanning mirror moved by a stepping motor, and reflected back to the self-focusing lens. This forms the scanning arm of the LCFS. The light of another arm is reflected by a mirror through a fiber in a specific length and forms the fixed arm of the LCFS. Finally, the signals in the two arms are reflected, coupled to the 3 dB coupler, and passed through the two arms of the coupler. The signals can then be detected by a photodetector (PD). A data acquisition system (AD) is needed, which is connected by a computer. The fibers used to configure the schematic were Corning single mode fibers (SMF28) and the reference and sensing fibers were Corning SMF G657A/B optical fiber with an improved macrobend performance.

**Figure 2.**
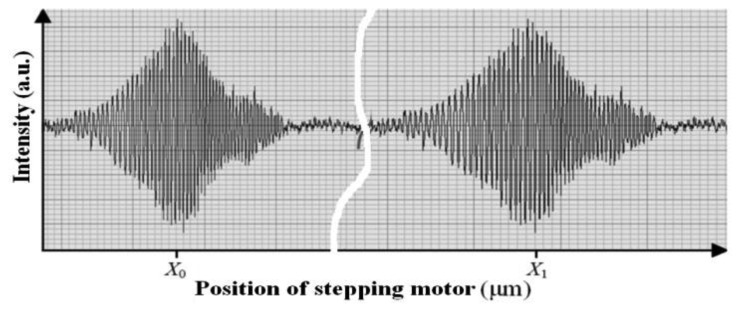
The interference fringes of LCFS and position reading.

Thus, the lengths of the reference fiber and sensing fiber, *l*_0_ and *l*_1_, can be configured in one scan range of the stepping motor to meet the two equal optical paths condition. When moving the stepping motor, the optical path difference (OPD) between the scanning arm and the fixed arm in the analyzer part will sequentially meet the equivalent OPD determined by the reference fiber and sensing fiber, respectively, in the sensing part and two peaks of the interferograms are obtained at different positions, *X*_0_ and *X*_1_, as showed in Figure 2. The sensing fiber was wound around the steel rebar in a number of circles and the reference fiber applied in the same way on a separate rebar. The rebar with the reference fiber was not connected to the electrical current and thus it only acted as a compensation for the corrosion sensor, while the rebar with sensing fiber was corroded by an electrochemical corrosion acceleration experiment. When the corrosion expansion increased, the length of the fiber *l*_1_ will be elongated to *l*_1_′. Then the stepping motor position *X*_1_ must be moved to *X*_1_′ to meet the equal optical path length condition. Thus, by reading the stepping motor position *X*_1_′ one can measure the length of the sensing fiber that is determined mainly by the corrosion expansion and a little from any non-corrosion-expansion factors. On the other hand, the reference fiber will not be sensitive to the corrosion expansion, because the current is not applied through the rebar with the reference fiber. It only measures the contributions from the non-corrosion-expansion factors, such as temperature expansion, *etc.* Thus, most of the fluctuations in the sensing fiber can be reduced based on the measurements of the reference fiber. Finally, the span between the two positions Δ*X*_0_ = |*X*_1_ − *X*_0_| can be recorded as an initial state.

In the corrosion monitoring experiments, when chloride ions penetrate the steel rebar across the concrete, corrosion happens, thus the steel expands because the rust product has a volume of about twice to quadruple that of the steel rebar it replaces when fully dense. As a result, the length of the sensing fiber will become *l*_1_′ in response to the corrosion expansion. The stepping motor position becomes *X*_1_′, whereas the reference fiber will suffer the fluctuation and reads as *X*_0_′. Now, the span, which is measured by reading the positions of the stepping motor, becomes Δ*X*_1_ = |*X*_1_′ − *X*_0_′| from the initial one Δ*X*_0_ = |*X*_1_ − *X*_0_|. The deformation of the sensing fiber, Δ*l*, caused by the corrosion expansion, can be calculated based on the equal OPD principle in Michelson configurations as [10]:

(1) $$\Delta l=\left|\Delta X_{1}-\Delta X_{0}\right|\cdot L_{N}/n$$

where, *n* is the effective refractive index of the sensing fiber, *L*_N_ = 1.25 μm is given by the specification of one step length of the stepping motor, |Δ*X*_1_ − Δ*X*_0_|·*L*_N_ is the distance measured by reading the positions of the stepping motor, and Δ*X*_0_ is the initial span before the corrosion experiment. And the corrosion expansion strain, *ε*, can be estimated as:

(2) $$\varepsilon =\Delta l/l_{1}$$

With the help of an effective signal processing [12,13], the readout of the interferogram peak for a deformation measurement can achieve the resolution of Δ = 5 μm. When the sensing fiber length is chosen as *l*_1_ = 3 m, the accuracy of the measurement on corrosion expansion strain could approach 2 με, which can be calculated as the minimum resolution: *ε*_min_ = Δ/(*nl*_1_) = 5/(1.46×3) = 1.14 με < 2 με.

### 2.2. The Principle of BOTDA

The fiber optic Brillouin sensing technique is based on the Brillouin scattering, which is a result of the interaction between incident lightwaves in an optical fiber and the acoustic phonons. The nature of the acoustic phonons is due to the interaction that causes periodic changes of refractive indices of the optical fiber. This periodic change basically acts as a Bragg grating that moves at acoustic velocity. The Brillouin scattered light is generated as a backscattered light that propagates in the direction opposite to the incident lightwaves if the Bragg condition is met. In addition, the backscattered light would have the incident frequency corresponding to the Bragg condition. When the grating is moving, the backscattered light exhibits a Doppler frequency shift. The Brillouin frequency shift *v*_B_, also called the Doppler frequency shift, is directly related to the local velocity of the acoustic waves, which depends on the local material temperature or strain, and it is given by [14,15]:

(3) $$v_{B}=2nV_{a}/\lambda_{0}$$

where, *V*_a_ is the acoustic velocity of the phonon, and *λ*_0_ is the wavelength of the incident light. Both *n* and *V*_a_ are strongly dependent on the temperature and strain applied on the sensing fiber. Thus, the Brillouin frequency shift, Δ*v*_B_, is dependent on temperature, *T*, and strain, *ε*, and can be expressed as [16]:

(4) $$\Delta v_{B}=v_{B}(\varepsilon ,T)-v_{B}(0,0)=C_{\varepsilon }\varepsilon +C_{T}T$$

where *C*_ε_ and *C*_T_ are the strain and temperature coefficients, respectively. *v*_B_(0,0) represents the Brillouin frequency without both temperature and strain applied. In this case, the temperature coefficient was ignored based on two treatments. One is to maintain the bath solution at constant temperature and the other is using a reference fiber as a cancellation. Therefore, we only considered the strain coefficient for corrosion expansion strain measurements that can be expressed as:

(5) $$C_{\varepsilon }=\Delta v_{B}/\varepsilon$$

This was usually calibrated by applying a specific strain and then calculating the strain coefficient based on the corresponding Brillouin spectrum obtained by an instrument. The data sheet provides a guide for users to choose the specification of the fiber as a sensing fiber.

### 2.3. The Relationship between BOTDA and LCFS

**Figure 3.**
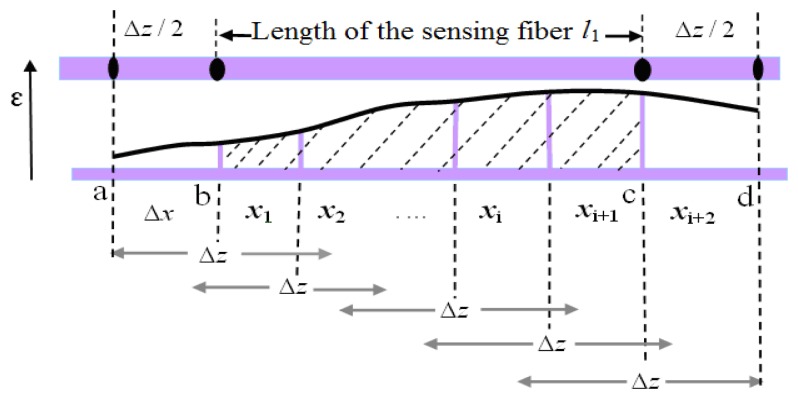
The distributed strain along with the sensing fiber.

LCFS can measure the total deformation of the whole sensing fiber. Meanwhile, BOTDA obtains the distributed strain over the spatial-resolution length along with the sensing fiber, as showed in Figure 3.

At the splices of the sensing fiber, there are at least half-length of spatial resolution, Δ*z*/2. The spatial resolution, Δ*z*, of BOTDA is given by [15]:

(6) $$\Delta z=\frac{c\tau }{2n}$$

where, *τ* is the duration of the incident optical pulse, *c* is velocity of the light wave propagated in vacuum. When *τ* = 10 ns was chosen, the spatial resolution can be estimated as Δ*z* ≈ 1 m. Based on Equation (2), the total deformation Δ*l* can be related to the distributed strains with the given length of the sensing fiber *l*_1_.

The strain is distributed along the sensing fiber, and the total deformation of steel can be deduced by the integral of these strains. Here, Δ*x* = 0.41 m determined by the sampling interval between the two points set up before the measurement from the panel, *x*_i_ are the sequential discrete points. In this corrosion expansion measurement, for the sensing fiber there is a trade-off between the spatial resolution and the bending loss. The 3 meters length is chosen for the 18 mm-diameter of the steel rebar based on trial and error experiments. Because the half length of the spatial resolution, Δ*z*/2, has been overlapped with the sensing fiber, the BOTDA could obtain the strain from the point, a, rather than from the start point b of *l*_1_. The same sensing mechanism happened at the end of the sensing fiber.

The methods developed to get rid of the limitations of the spatial resolution are an interesting topic [17–20]. Much progress has been reported in high-resolution, long-range schemes, by which Brillouin scattering-based technique was extended, such as random-access Brillouin sensors based on radar-inspired technique [21] and pre-pump Brillouin technique [22], *etc.* Here, we only directly considered the spatial resolution by an equivalent sensing fiber length *l*_1_ + Δz in Equation (2). Thus, a relationship between the total deformation, Δ*l*, and the integral of the distributed strain measured by BOTDA, *ε*(*x*), can be set up as:

(7) $$\Delta l=\int_{0}^{l_{1}+\Delta z}\varepsilon (x)dx$$

In the BOTDA system, ε(*x*) is just recorded at discrete points, such as *ε*(*x*_i_), and Equation (7) will be changed to be the following equation:

(8) $$\Delta l=\Delta x\sum_{i=1}^{N}\frac{\varepsilon (x_{i})+\varepsilon (x_{i+1})}{2}+(l_{1}-N\cdot \Delta x)\frac{\varepsilon (x_{N+1})+\varepsilon (x_{N+2})}{2}+\frac{\varepsilon (x_{1})+\varepsilon (x_{N+2})}{2}\Delta z$$

where *N* = int [*l*_1_/Δ*x*], especially, when the strain is uniform, that is to say *ε*(*x*) = *ε*_0_, the Equation (7) or (8) will become Δ*l* = *ε*_0_(*l*_1_ + Δ*z*), and this is the form presented in Equation (2). This will be well matched by the integral of the shadowed area in Figure 3 and should approximate the total corrosion expansion deformation of the steel along with the sensing fiber.

## 3. Experiments

### 3.1. Corrosion Sensor

**Figure 4.**
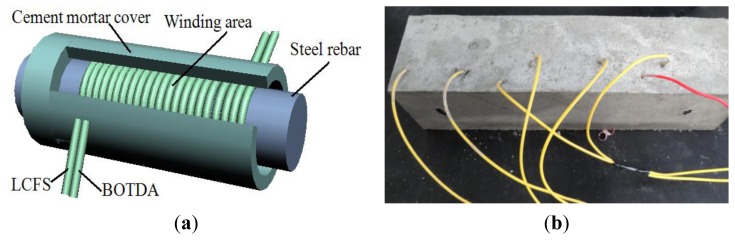
The schematic of the sensing fibers inside the RC and the experimental specimen. (**a**) Fibers wound around the steel rebar directly; (**b**) The specimen for steel corrosion.

After being polished, the diameter of the steel rebar was 18 mm and the length was 18 cm. Two pieces of the Corning SMF G657A/B optical fibers, which have a minimum bending radius of 7.5 mm and in 3 meters length, were directly wound around the rebar in a number of circles next to each other, one for LCFS and the other for BOTDA, as shown in Figure 4a. The strain coefficient of the fiber was calibrated as 480 MHz/% strain [8]. About 9 mm-thick cement mortar cushion layer was coated to the steel rebar and formed a solidification pillar. The introduced fibers were armored for protection as shown in Figure 4b.

The corrosion expansion sensor was placed along with the rebar in the specimen. The reference fiber was wound and put at certain distance from the corrosion environment to avoid the corrosion expansion. The size of the concrete specimen was 100 mm × 100 mm × 300 mm. The ultimate strength of the concrete was approximately 30 Mpa. The cement was P.O. 32.5 and the cement, water, sand, and aggregate mass ratio was 1:0.53:1.64:3.49. After completed the casting, the specimen was cured for 7 days using the steam curing method, and then kept at room temperature (22 °C) for 3 days before doing the experiments.

### 3.2. Accelerating Corrosion Experiment

**Figure 5.**
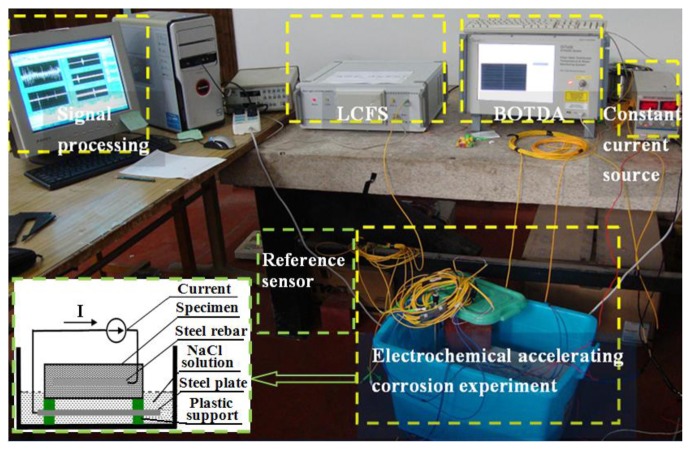
Schematic of the electrochemical corrosion acceleration experiment system.

An electrochemical corrosion experiment was set up to accelerate the corrosion process of steel rebar in the RC specimens, as shown in Figure 5. The concrete specimen with the sensing fiber was put on the plastic support and 1/5 of the specimen was in the NaCl solution with a mass concentration of 5.0%. Whereas the position embedded the steel rebar was above the solution as shown in the inset of Figure 5. There was a stainless steel plate at the bottom of the bath container.

Before the experiments being done, the concrete specimen was placed in the bath solution for one day, and then a constant current was applied from the rebar to the plate to connect the bath solution and formed a 40 mA electrical current. Thus the steel rebar acted as an anode and the stainless steel plate functioned as a cathode. On the other hand, the specimen with the reference fiber was not connected to the electrical current and only put on near the corrosion specimen. The experiments were carried out in a room at a constant temperature of 22 °C.

## 4. Results and Discussion

The electrochemical corrosion acceleration experiment was studied on the RC structure specimen. When the specimen experiment lasted for 130 h, the BODTA signal became too weak to be detected, while the deformation monitored by the LCFS could get the data until 260 h.

**Figure 6.**
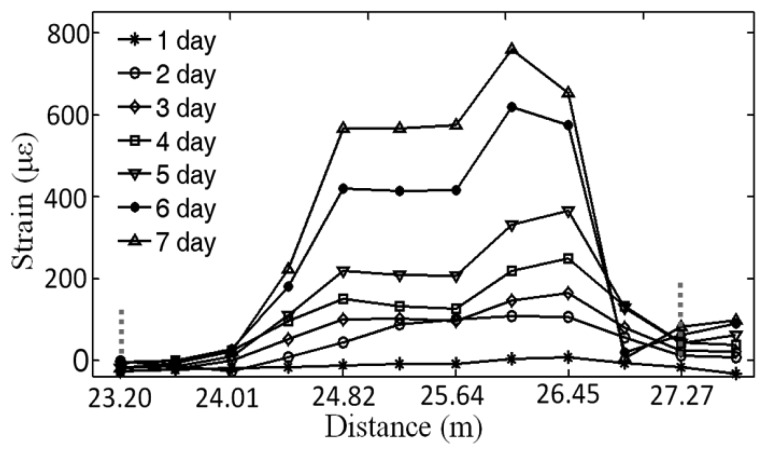
Distributed strain caused by the corrosion expansion was monitored by BOTDA within 7 days corresponding to the 3-meter length of the sensing fiber.

Figure 6 showed a corrosion expansion distributed strain monitored by the BOTDA for the specimen at the distance of introduction fiber between 23.20 and 27.27 m, which was indicated by the two vertical dashed-gray lines and corresponding to the 3 meter length of the sensing fiber, *l*_1_, plus the spatial resolution, Δ*z*, *i.e*., *l*_1_+Δ*z* ≈ 4.0 m. The curves from 1 day to 7 day were presented the increasing of the distributed strains within 7 days, respectively.

**Figure 7.**
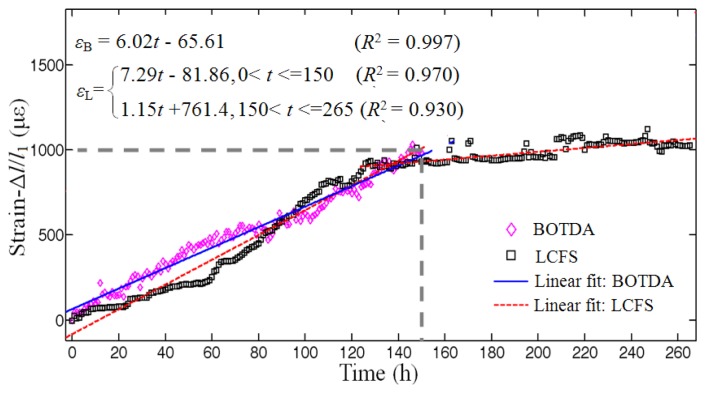
Comparison on the total strain over the sensing fiber of the specimen between the integrated results from the data obtain by the BOTDA and these measured by the LCFS in the electrochemical corrosion acceleration experiment.

We integrated the area under the curves of 7 days, separately, corresponding to the distance as described by Equation (7) and shown in Figure 7, corresponding to the Figure 6.

The data from the BOTDA illustrated that the total deformation (presented as the total strain along with the sensing fiber) increased steadily and the degree of corrosion grew gradually over time.

Figure 7 shows the strain monitored from the specimen. The rectangular dotted line was the data measured by the LCFS and the diamond dotted line was the results calculated from the BOTDA. The lines had a matching tendency within 150 h of corrosion experiment. After the corrosion time of 150 h the BOTDA signal was too weak to be detected, whereas the LCFS reading lasted to about 265 h, and the slope of the fit line of the dada of LCFS was almost horizontal, even if the strain was steady around 1,000 με. At this time, the rust spilled out, the LCFS result decreased and some strain fluctuations were observed.

The diameter of the steel rebar was 18 mm, and then its perimeter was about 18π ≈ 0.056 m. The distance resolution of the BODA was 0.41 m, which was determined by the sampling rate of the analog-digital-convertor. This made the BOTDA record only one datapoint every 0.41/0.056 = 7 circles of the sensing fiber around the steel rebar. Hence, the reason should be due to the fact that the read out strains by the BOTDA was the convolution results between the sensing fiber length and the spatial resolution [17,18]. Thus it was also a kind of averaged strain. On the other hand, the LCFS accumulated all the strains, no matter whether sharp or not, as the total deformation of the sensing fiber, so these two sensors matched well the early stages of the corrosion experiment.

**Figure 8.**
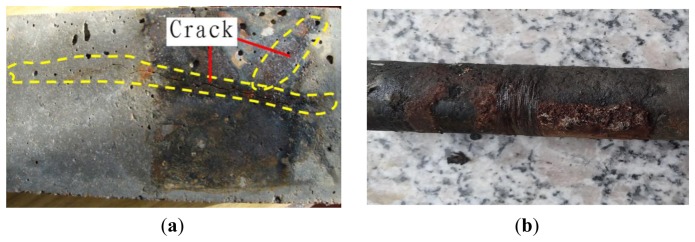
The samples after the corrosion experiments. (**a**) The cracks found outside of the specimen after the experiment; (**b**) Surface of the steel rebar after the corrosion experiments.

After the experiments, some cracks were found at the bottom of the specimen, as shown in Figure 8a. The longest of the crack reached about 100 mm, whereas the others were around 40 mm. The position of the rust spilled out of the specimen was close to the sensing fiber wound. The steel rebar with the sensing fiber were taken out from the specimen after the experiments, as shown in Figure 8b. The rebar was severely corroded and obvious rust was distributed between the steel and sensing fiber winding places.

Based on our five repeated experimental results, the BOTDA measurements were indeed restricted to about 1,000 με due to the fact the signal was too weak to be detected. Combined with our previous experience on the corrosion monitoring, which was published [8] the maximum strain can reach 5,000 με by using the same Corning SMF G657A/B sensing fiber in the same BOTDA system. The difference between these two usages was the sensor fabrication. In [8], we put the sensing fiber on a layer of a cement mortar cushion, which mediated the strain from the steel rebar to the sensing fiber coil, but the sensing fiber was wound on the steel rebar directly in this work. This must be the reason that the rust causes too much bending in the sensing fiber. This will reduce the forward light power in a large part and, therefore, the corresponding backscattering signal will suffer a fundamental attenuation.

## 5. Conclusions

This study aimed to utilize a LCFS method to survey the performance of BOTDA sensors, which have been employed to monitor the corrosion procedure occurring in RC structures. The corrosion experiments with 18 mm-diameter steel rebar demonstrated that the BOTDA method could be employed to monitor the corrosion inside the structure in the early stage, when the expansion strain was less than 1,000 με, and the results can be explained well by the strain measured directly by the LCFS. The LCFS method can be used for monitoring the corrosion expansion strain from the beginning of the corrosion to the destruction of the structure. The further experiments will be designed to find out the models of the cracks generation based on both the BOTDA and the LCFS techniques.
